# Biomedical Application of Functional Materials in Organ-on-a-Chip

**DOI:** 10.3389/fbioe.2020.00823

**Published:** 2020-07-22

**Authors:** Chizhu Ding, Xiang Chen, Qinshu Kang, Xianghua Yan

**Affiliations:** ^1^State Key Laboratory of Agricultural Microbiology, College of Science, Huazhong Agricultural University, Wuhan, China; ^2^State Key Laboratory of Agricultural Microbiology, College of Animal Sciences and Technology, Huazhong Agricultural University, Wuhan, China; ^3^The Cooperative Innovation Center for Sustainable Pig Production, Wuhan, China; ^4^Hubei Provincial Engineering Laboratory for Pig Precision Feeding and Feed Safety Technology, Wuhan, China

**Keywords:** organ-on-a-chip, microfluidics, elastomer, hydrogel, microfabrication

## Abstract

The organ-on-a-chip (OOC) technology has been utilized in a lot of biomedical fields such as fundamental physiological and pharmacological researches. Various materials have been introduced in OOC and can be broadly classified into inorganic, organic, and hybrid materials. Although PDMS continues to be the preferred material for laboratory research, materials for OOC are constantly evolving and progressing, and have promoted the development of OOC. This mini review provides a summary of the various type of materials for OOC systems, focusing on the progress of materials and related fabrication technologies within the last 5 years. The advantages and drawbacks of these materials in particular applications are discussed. In addition, future perspectives and challenges are also discussed.

## Introduction

An organ-on-a-chip (OOC) is a microfluidics-based cell culture device that contains continuously perfused chambers inhabited by living cells to simulate tissue- and organ-level physiology (Bhatia and Ingber, 2014; Ahadian et al., 2018). The development of OOC stems from the recognition that the conventional two-dimensional static cell culture methods lack the ability to mimic the environment that cells experience *in vivo* (Ryan et al., 2016; Duval et al., 2017). Microfluidic technology provides a way to simulate spatiotemporal chemical gradients, dynamic mechanical forces, and critical tissue interfaces by manipulation of fluids at micro levels. OOC systems that can recreate key aspects of the complex physiological microenvironment of human lung (Huh et al., 2010), heart (Maoz et al., 2017), stomach (Lee K. K. et al., 2018), intestine (Kim et al., 2016), liver (Weng et al., 2017), kidney (Sateesh et al., 2018), blood vessels (Wang et al., 2015), etc., have been developed. Moreover, multi-organs-on-a-chip or body-on-a-chip systems have been proposed (Sung et al., 2019; Zhao et al., 2019a). OOC platforms have shown application potential in a lot of biomedical fields such as fundamental physiological and pharmacological researches (Zhang and Radisic, 2017; Zhang et al., 2018a).

Materials play the major roles in the development of microfluidics and OOC technologies. In general, material considerations include non-toxic to cells, gas permeable, optically transparent for microscopic imaging, costs of the materials and the fabrication process, and the ability to model specific properties of organs (Lee et al., 2014). Although polydimethylsiloxane (PDMS) is still the most common material for laboratory research, emerging materials such as hydrogel, paper and hybrid materials are being developed and used. In this mini review, the classic and advanced materials and fabrication technologies for OOC devices are introduced and discussed, focusing on the progress within the last 5 years. The major properties, limitations, and typical applications in OOC of some representative materials are summarized in Table 1. Future perspectives and challenges in the development of materials for OOCs are briefly discussed.

**TABLE 1:** Typical materials for OOC applications.

Materials	Major properties	Limitations	Typical applications in OOC
Glass	+ Surface stability + Optically transparent + Electrically insulating	– Not gas permeable – High cost of fabrication	• OOC device substrate • Glass-based chip for transform studies (Kulthong et al., 2018) • Enabling real-time imaging (Li X. et al., 2018)
PDMS	+ High elasticity + High gas permeability + Biocompatibility + Rapid prototyping	– Hydrophobicity – Strong adsorption of biomolecules – Not compatible with organic solvents	• Most common OOC substrate • Biomimetic cell culture scaffold (Kim et al., 2012) • Microvascular model (Zhang W. et al., 2016)
Plastic	+ Optically transparent + Low absorption + Rigid + Suitable for mass production	– Less gas-permeable – Unsuitable for prototyping	• OOC device substrate (Miller and Shuler, 2016) • Porous membrane to model tissue-tissue interfaces (Pocock et al., 2017)
Paper	+ Highly porous + Matrix of cellulose + Potable and low cost	– Limited detection methods – Difficult to integrate microcomponents	• OOC device substrate • TRACER (Young et al., 2018) • Model of respiratory system (Rahimi et al., 2016)
Collagen	+ Biocompatible + Enzymatically degradable + Similar in structural and mechanical properties to native tissues + Good cell adhesion	– Weak mechanical properties	• Microvascular networks (Zheng et al., 2012) • Scaffold mimicking 3D villi structure (Shim et al., 2017) • Neurovascular model (Adriani et al., 2017) • Skin model (Lee S. et al., 2017) • Kidney model (Lee S. J. et al., 2018) • Pumping heart chamber model (Li R. A. et al., 2018) • Liver spheroids, tumor spheroids (Yamada et al., 2015; Jeong et al., 2016)
Gelatin	+ Biocompatible + Biodegradable + Similar in composition to collagen + Good cell adhesion + Tunable properties by the addition of functional group (e.g., GelMA)	– Weak mechanical properties – Rapid degradation	• Heart-on-a-chip (Zhang Y. S. et al., 2016) • Skin model (Zhao et al., 2016) • Microvascular networks (Yang et al., 2016) • Spheroid-based liver model (Bhise et al., 2016)
Alginate	+ Biocompatible + Biodegradable + Easy functionalization + Immediate gelation at mild condition	– Weak mechanical properties – Poor cell adhesion – Uncontrollable degradation	• Scaffolds containing living cells (Ning et al., 2016) • Liver spheroids, tumor spheroids (Chan et al., 2016; Kang et al., 2016) • Hydrogel fibers (Zhu et al., 2017)
PEG and its derivatives (e.g., PEGDA)	+ Biocompatible + Tunable and precise mechanical and degradation properties + Relatively low protein adsorption	– Less cell adhesive – Limited biodegradation	• Self-organizing cardiac microchambers (Ma et al., 2015) • Liver organoids generation (Ng et al., 2018) • Intestinal organoids generation (Cruz-Acuña et al., 2017)

## Materials for OOCs

### Inorganic Materials

Silicon and glass are the main inorganic materials for OOCs. The first-generation microscale cell culture analog (μCCA) devices mimicking the organ-level function of human physiology were fabricated on silicon (Sin et al., 2004; Mahler et al., 2009). Compared to opaque silicon, glass is optically transparent and optimal for real-time imaging, while reducing the absorbance of hydrophobic molecules and the adsorption of biomolecules (Lee S. et al., 2017; Kulthong et al., 2018). Nevertheless, glass chips with enclosed channels are not suitable for long-term cell culture because glass is not gas permeable. Another problem is that glass is typically processed with standard photolithography and etching, which are time-consuming and expensive. Recently, femtosecond laser ablation technique has been applied to fabricate 3D structures in glass-based OOCs (Xu et al., 2015; Schulze et al., 2017). Liquid glass, a photocurable amorphous silica nanocomposite enabling soft replication, has been developed for low-cost prototyping of glass microfluidics (Kotz et al., 2016).

### Elastomer

**FIGURE 1:**
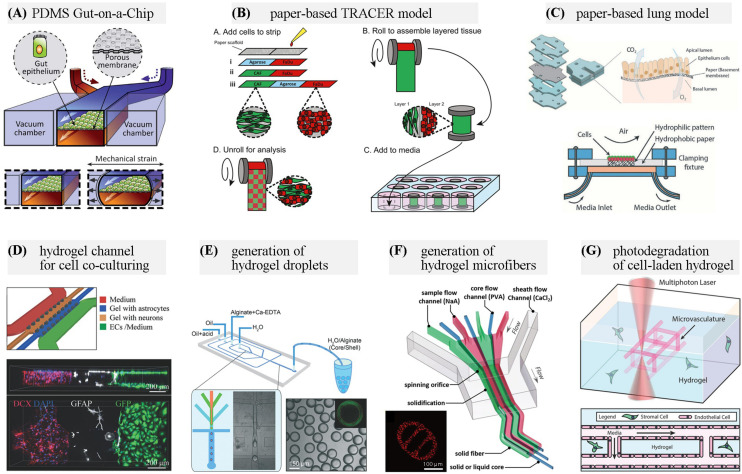
Representative materials used for OOC applications. **(A)** A PDMS gut-on-a-chip device containing two microchannels separated by a flexible porous membrane, and vacuum chambers on both sides. Adapted with permission from Kim et al. (2012), The Royal Society of Chemistry. **(B)** Configuration of a paper-based Tissue Roll for the Analysis of Cellular Environment and Response (TRACER) platform. Adapted with permission from Young et al. (2018), Elsevier. **(C)** Paper-based air-liquid-interface for *in vitro* human respiratory system model. Adapted with permission from Rahimi et al. (2016), The Royal Society of Chemistry. **(D)** A neurovascular chip with gel channels for cell co-culturing. Adapted with permission from Adriani et al. (2017), The Royal Society of Chemistry. **(E)** Generation of core-shell hydrogel droplets for spheroid-based liver model. Adapted with permission from Chen Q. et al. (2016), The Royal Society of Chemistry. **(F)** On-chip spinning of hydrogel microfibers with morphological and structural complexity, as well as a heterogeneous composition. Adapted with permission from Yu et al. (2016), John Wiley & Sons. **(G)** Microchannel generation within cell-laden hydrogel by user-programmed multiphoton excitation induced localized degradation. Adapted with permission from Arakawa et al. (2017), John Wiley & Sons.

Elastomers are polymers with elasticity, and generally having lower Young’s modulus and higher yield strain than other materials. PDMS is one of the most common materials used for the fabrication of microchips for the life science applications. It is not only gas permeable, biocompatible and optically transparent, but also particularly useful in prototyping new devices by soft lithography and micromolding technique (McDonald and Whitesides, 2002). Its elasticity allows to demold the PDMS replica with complex 3D structures (Suzuki et al., 2017). Moreover, the elasticity can be used to fabricate biomimetic cell culture scaffolds, such as the human lung-on-a-chip and gut-on-a-chip with pneumatically controlled deformation (Figure 1A) (Huh et al., 2010; Kim et al., 2012) and the microvascular models (Choi et al., 2014; Zhang W. et al., 2016). Apart from the conventional replication method, other strategies including hybrid stamp approach (Kung et al., 2015), razor-printing (Cosson et al., 2015), sacrificial template methods (Cheng et al., 2016) can also be used for PDMS. An optimized blend of PDMS-methacrylate macromers has been developed and demonstrated for 3D stereolithography (SL) with mechanical properties similar to conventional thermally cured PDMS. The 3D-printable PDMS resin would facilitate the fabrication of PDMS-based OOC platforms (Bhattacharjee et al., 2018).

Nevertheless, some characteristics of PDMS such as incompatibility with organic solvents, hydrophobicity and strong adsorption of biomolecules also limits its application in certain fields. Surface modifications of PDMS or the use of alternative materials may be feasible solutions. Some polymers with similar fabrication procedures suitable for rapid prototyping, higher rigidity, and better resistance to solvents, such as thermoset polyester (TPE), polyurethane methacrylate (PUMA) and Norland adhesive 81 (NOA81), have been assessed as complementary to PDMS (Sollier et al., 2011). However, they have not developed into the common choice in OOC devices. Styrene-(ethylene/butylene)-styrene (SEBS) copolymer (Domansky et al., 2017) and tetrafluoroethylene-propylene (FEPM) elastomer (Sano et al., 2019) that do not absorb hydrophobic molecules have been used for fabrication of OOCs for drug discovery and development.

To establish vascular networks, a biodegradable elastomer, poly(octamethylene maleate (anhydride) citrate) (POMaC) is used to construct a scaffold (AngioChip) with a build-in microchannel network. This material provides desired mechanical properties, biodegradation rate, and biocompatibility for specific applications (for example, human myocardium or liver tissue engineering) (Zhang B. et al., 2016; Zhang et al., 2018b). In a platform termed Biowire II, two parallel POMaC wires are suspended in the microwell between which cardiac tissue would self-assemble, matching the mechanical properties of the native cardiac tissue (Zhao et al., 2019b). A biodegradable elastomer with significantly low Young’s modulus has been synthesized and demonstrated utility in cardiac tissue engineering constructs (Davenport Huyer et al., 2016).

### Plastic

Typical plastic materials for microfluidics include poly(methyl methacrylate) (PMMA), polycarbonate (PC), polystyrene (PS), Cyclic Olefin Polymer (COP) and Cyclic Olefin Copolymer (COC). They are generally optically transparent, more rigid than elastomers, less gas-permeable than PDMS, resistant to the permeation of small molecule, but incompatible with most organic solvents (Ren et al., 2013; Gencturk et al., 2017). Among these materials, PMMA has been widely utilized as substrate materials for OOC devices due to its rigid mechanical property, excellent optical transparency and low auto-fluorescence background (Chen X. et al., 2016; Miller and Shuler, 2016). Porous PC membranes are usually incorporated between microchannels in OOC systems to model tissue-tissue interfaces (Shah et al., 2016; Pocock et al., 2017). PS is highly biocompatible and suitable for cell growth and adhesion (Lee et al., 2019). COP and COC present excellent optical transmittance in both the visible and UV range, allowing for high quality fluorescence imaging. They are also FDA approved, showing a promising potential for future routine clinical use (Mottet et al., 2014). And recently, polylactic acid (PLA) as a sustainable, low absorption, low autofluorescence alternative to other plastics for OOC applications has been demonstrated (Ongaro et al., 2020).

Thermoplastics are suitable for thermo-processing, which is excellent for commercial production due to high production-rate and low cost, but not economical for prototypic use (Ren et al., 2013). Some novel materials such as a photocurable soft lithography compatible liquid PS prepolymer (Nargang et al., 2014) and a fast curing PMMA prepolymer that can be used as a negative photoresist and directly structured using UV or visible light (Kotz et al., 2018) have been developed for rapid prototyping. Fabrication methods for rapid prototyping of whole-thermoplastic microfluidic chips with microvalves and micropumps are being developed and could be employed for the OOC applications (Pourmand et al., 2018; Shaegh et al., 2018).

### Paper

Paper microfluidics has the advantages of lightweight, easy-of-use and low cost. The cellulose matrix of paper allows for a porous structure for cell growth in a 3D format. Paper-based microfluidics with dynamic control of physiological microenvironment can be formed on multilayered paper and be used as high-throughput test platforms (Mosadegh et al., 2015; Sapp et al., 2015). And by directly incorporating a luminescent sensing film, the spatiotemporal oxygen consumption rates or pH gradients can be monitored in real-time through quantitative image analysis (Boyce et al., 2016; Kenney et al., 2018). In a device named tumor roll for analysis of cellular environment and response (TRACER), different cells are seeded in a defined area on the paper, and then the 3D tumors are assembled by rolling the biocomposite strip. By unrolling the strip, the model can be rapidly disassembled for snapshot analysis (Figure 1B) (Rodenhizer et al., 2016; Young et al., 2018). The Khademhosseini group presented the use of hydrophobic paper as a semi-permeable membrane for culturing cells at the air-liquid interface. The final paper-based device provides a cost-effective platform for human respiratory system studies under physiologically relevant conditions (Figure 1C) (Rahimi et al., 2016).

Having many similarities with paper, nitrocellulose membranes (Guo et al., 2018), threads (Yang et al., 2014), and cloths (Wu and Zhang, 2015) have also been investigated as a scaffold for cell culture. They have potential as superior alternatives to paper due to the stronger, higher controllable rates for fluid mixing and lower environmental impact (Bagherbaigi et al., 2014).

### Hydrogel

Hydrogels are polymeric materials distinguished by high water content (Seliktar, 2012). They can mimic salient elements of native extracellular matrices (ECMs) due to their high biocompatibility and tunable properties, such as elasticity, porosity, permeability, stiffness and degradability. These properties of hydrogels are largely dependent on the types, gelation methods, and fabrication technologies. Hydrogels can be broadly classified into natural, synthetic, and hybrid according to their source (Caliari and Burdick, 2016; Jiang et al., 2016; Liu et al., 2019). Typical natural hydrogels include collagen, alginate, gelatin, agarose, and fibrin. They are generally highly biocompatible and containing cell-binding sites for cell attachment, spreading, growth, and differentiation. Collagen is the most common ECM component in the body and one of the most widely used hydrogels for bioengineered tissue microenvironments (Antoine et al., 2014). Gelatin has a similar composition to collagen. Gelatin methacryloyl (GelMA) hydrogels closely resemble some essential properties of native ECM and can be microfabricated using different methodologies (Yue et al., 2015). In recent studies, ECM hydrogels derived from decellularized tissues have been used to provide a supportive microenvironment capable of long-term culture of islets or directing cell growth (Giobbe et al., 2019; Jiang et al., 2019). Nevertheless, natural hydrogels suffer from some drawbacks such as relatively poor mechanical properties, limited long-term stability, and batch-to-batch variability. Typical synthetic hydrogels include polyethylene glycol (PEG) and its derivatives [e.g., PEG-diacrylate (PEG-DA)], polylactic acid (PLA), poly(lactic-*co*-glycolic acid) (PLGA), and poly(ε-caprolactone) (PCL). Synthetic and hybrid hydrogels are more tunable to provide desired mechanical properties and degradation rates.

Since the materials for chip fabrication mentioned above, such as PDMS and plastics, are unfavorable for cell attachment, hydrogels are often coated on the channel surfaces or integrated into OOC devices (Annabi et al., 2013; Zhang X. et al., 2016). By incorporating a collagen scaffold that mimics the human intestinal villi, the microfluidic gut-on-a-chip could provide cells with both 3D tissue structure and fluidic shear to induce further improvement in gut functions (Kim et al., 2014; Shim et al., 2017). A hydrogel microlayer consisting of type I collagen and Matrigel has been used in a lung airway-on-a-chip, replacing the semipermeable, PDMS membrane, allowing for the co-culture of epithelial cells and smooth muscle cells (Humayun et al., 2018). Hydrogels can also act as diffusion barriers which separate cells while permitting the soluble factors, such as nutrients, proteins, and signaling molecules exchange (Figure 1D) (Adriani et al., 2017). Compared with other artificial membranes used in OOC models, hydrogel barriers allow close cell association by making direct cell–cell contact between multiple cell types possible (Tibbe et al., 2018). Thanks to the progress in 3D (bio)printing technology, cell-laden hydrogels scaffolds can be rapidly created with spatial heterogeneity in predefined patterns (Miri et al., 2018; Moroni et al., 2018). Methods for the fabrication of hydrogel-based microfluidic chips are being developed. By stereolithographic high-resolution printing of PEG-DA, microfluidic chips with biofunctionalized complex 3D perfusion networks can be rapidly fabricated (Zhang and Larsen, 2017). Combining casting and bonding processes, Nie et al. (2018) fabricated a hydrogel-based vessel-on-a-chip of gelatin and GelMA.

Another frequently employed strategy for cell-based assays using hydrogels is to generate cell-encapsulated hydrogel droplets or hydrogel microfibers, especially through microfluidic approaches. On-chip production, storage, sorting and high-resolution imaging of hydrogel droplet has been achieved (Aubry et al., 2015). The generated microgels, including multicellular microspheres and microcapsules, create microenvironments for cell growth and proliferation (Figure 1E) (Headen et al., 2014; Alessandri et al., 2016; Chen Q. et al., 2016). By adjusting flow conditions in the microfluidic devices, various microfibers with morphological and compositional diversity can be generated as platforms for cell coculture (Figure 1F) (Yu et al., 2016; Xu et al., 2017; Liu et al., 2018; Xie et al., 2018).

The development of “smart” responsive hydrogels adapting to external stimuli has found its applications in OOC. Light-responsive hydrogels are of particular interest because of their capability of contact-free remote manipulation and the inherent space-time control capabilities of light stimulation (Li et al., 2019). Softening or stiffening hydrogels can be achieved by sequential photodegradation and photoinitiated crosslinking reactions, which is useful to design dynamic cell microenvironments (Rosales et al., 2017). Based on photodegradable hydrogels, 3D vascular networks within hydrogels can be altered dynamically, permitting user-defined 4D control even in the presence of live cells (Figure 1G) (Arakawa et al., 2017). In addition to applications related to cell culture, nanocomposite hydrogels crosslinking with metal or metal-oxide nanoparticles, and hydrogels of conducting polymers, such as poly(3,4-ethylenedioxythiophene): poly(styrene sulfonate) (PEDOT:PSS) based hydrogels, have biocompatibility, desired electrical and mechanical properties, and can be used to make sensors integrated in OOC platforms (Gaharwar et al., 2014; Park et al., 2015; Lu et al., 2019).

### Organic-Inorganic Hybrid Materials

Organic–inorganic hybrid materials offer the advantages of the organic content and the inorganic matrix. By combining inorganic clay nanoparticles with polymer matrix, clay-polymer nanocomposites has the ability to marry important biomaterial parameters such as porosity or self-organization with mechanical strength and toughness. Enhancements in cell adhesion, proliferation, and differentiation in response to clay nanoparticles have been observed in investigation into clay-cell interactions, suggesting the potential for the generation of multifunctional scaffolds for tissue engineering (Dawson and Oreffo, 2013). A UV-curable hybrid ceramic polymer Ormocomp is inherently biocompatible supporting cell adhesion without any additional coating and has been utilized as scaffolds for cell culture (Scheiwe et al., 2015; Järvinen et al., 2020). Ormocomp has excellent transparency for VIS and near UV down to 350 nm. In a recent study, round concave cross-sectional shaped microchannels of Ormocomp were fabricated via single step lithography to improve the sensitivity of fluorescence imaging (Bonabi et al., 2017). Novel organic-inorganic hybrid materials can potentially be used in the fabrication of OOC devices (Mechref et al., 2016a, b).

## Summary and Outlook

The OOC technology has been utilized in biomedical fields and has displayed great potential to speed up and simplify fundamental physiological and pathophysiological researches. The choice of chip materials is the first and crucial step for a successful OOC application. PDMS and plastics have been utilized as substrate materials for the majority of OOC platforms. Hydrogel materials are particularly suitable for mimicking native ECMs, and are often combined with other substrate materials to form hybrid chips. Many materials suitable for 3D (bio)printing technologies have been developed, providing a convenient method for prototyping complex chip structures. In particular, novel multi-material bioprinting technologies facilitate the fabrication of cell-laden constructs that highly similar to the biological tissues. These advances in materials and fabrication technologies have promoted the development of OOCs.

However, limitations and challenges exist. The hydrogel simulated microenvironments still differ from the native ECM microenvironments in stiffness, permeability and biochemical components. Moreover, the native microenvironment is diverse and may dynamically change during the stages of growth. It is important to design materials that can mimic the real ECM microenvironments as well as simple but precise methods to regulate the properties. In addition, the design of most OOC devices typically requires the assembly of hybrid materials. Novel materials together with fabrication methods covering both biological and engineering aspects can be a great challenge and an active area of research.

## Author Contributions

CD and XY conceived and designed the manuscript. CD and XC wrote the original draft. QK and XY revised the manuscript. All the authors contributed to the article and approved the submitted version.

## Conflict of Interest

The authors declare that the research was conducted in the absence of any commercial or financial relationships that could be construed as a potential conflict of interest.
